# Utility of p16INK4a expression for the interpretation of uterine cervical biopsies in Kenya

**DOI:** 10.11604/pamj.2021.40.55.21116

**Published:** 2021-09-22

**Authors:** Thierry Zawadi Muvunyi, Eliane Rohner, Siobhan O'Connor, Ahmed Yakub Kalebi, Wairimu Waweru, John Kairu, Willis Ochuk, Jennifer Susan Smith, Lucy Wangari Muchiri

**Affiliations:** 1 Department of Pathology, University of Nairobi, School of Health Sciences, Nairobi, Kenya,; 2 Department of Epidemiology, Gillings School of Global Public Health, University of North Carolina, Chapel Hill, NC, USA,; 3 Department of Pathology and Laboratory Medicine, University of North Carolina at Chapel Hill, Chapel Hill, NC, USA,; 4 Pathologists Lancet, Nairobi, Kenya,; 5 Lineberger Comprehensive Cancer Center, University of North Carolina, Chapel Hill, NC, USA

**Keywords:** Cervical biopsy, cervical intraepithelial neoplasia, dysplasia, hematoxylin and eosin, p16INK4a immunohistochemistry

## Abstract

**Introduction:**

histologic interpretation of hematoxylin and eosin-stained cervical biopsies is subject to substantial discordance among pathologists. Immunohistochemical staining for p16INK4a can reduce inter-observer disagreement. We did a cross-sectional study to evaluate the utility of p16INK4a staining in the assessment of cervical biopsies in Nairobi, Kenya.

**Methods:**

hematoxylin and eosin-stained sections from 91 colposcopic biopsies diagnosed as negative for dysplasia or as cervical intraepithelial neoplasia (CIN) grade 1-3 from 2011-2013 in Nairobi, Kenya, were reviewed and immunostained for p16INK4a. Agreement in interpretation of cervical biopsies was compared between primary and consensus review results.

**Results:**

on primary evaluation, 16 cases were negative for squamous dysplasia; 23 were CIN 1; 37 CIN 2; and 15 CIN 3. On consensus review, 32 cases were negative for dysplasia; 19 were CIN 1; 16 CIN 2 and 24 CIN 3. Agreement was moderate between primary and consensus histology review results for the diagnosis of low-grade versus high-grade squamous intraepithelial lesions (Kappa = 0.568). None of the cases negative for dysplasia were positive for p16INK4a expression, but in primary and consensus review results, 17% and 5% cases of CIN 1; 49% and 69% of CIN 2, and 80% and 96% of CIN 3 were p16INK4a positive, respectively.

**Conclusion:**

there was significant variability in the interpretation of cervical biopsies on hematoxylin and eosin between primary and consensus review assessments. 75% of CIN 1 cases that were upgraded to CIN 2 during consensus review expressed p16INK4a. These findings demonstrate the role of p16INK4a in increasing diagnostic accuracy and as a marker of high-grade CIN 2/3.

## Introduction

Cancer of the cervix is characterized by a marked variation in geographic distribution, with more than 85% of the global burden occurring in low- and middle-income countries [1]. In Kenya, the age-adjusted cervical cancer incidence rate is estimated at 34 per 100,000 person-years in 2018 [2], with an estimated 3,286 associated deaths. The natural history preceding cancer of the cervix has a long premalignant period that provides the opportunity for screening and treatment before progression of cervical precancer to cervical cancer [3]. Screening methods include Papanicolaou (Pap) smear, visual inspection, human papillomavirus deoxyribonucleic acid (DNA) testing, among others, followed, if available, by diagnostic confirmation with colposcopy and biopsy. Treatment plans are often established based on hematoxylin and eosin-stained cervical biopsy tissue: cervical intraepithelial neoplasia grade 1 is treated conservatively and followed up with screening in many countries, while high-grade cervical intraepithelial neoplasia grade 2 and 3 require further intervention and treatment. However, the diagnostic interpretation of hematoxylin and eosin-stained cervical tissue is subject to substantial discordance among pathologists, despite strict criteria for the diagnosis of cervical intraepithelial neoplasia [4-6]. As such, the impact of an inaccurate pathology diagnosis on patient management may be significant.

Immunohistochemical staining for p16INK4a has been shown to improve the inter-observer reproducibility of histologic diagnoses of cervical intraepithelial neoplasia [7]. Overexpression of p16INK4a in the cervix acts as a marker of the oncogenic activity of high-risk human papillomavirus infection [8]. Therefore, p16INK4a immunohistochemical staining can reduce both false-negative and false-positive biopsy results, and significantly improve the accuracy of cervical pre-cancer histologic diagnoses [7,9]. Additionally, patients with p16INK4a negative cervical intraepithelial neoplasia grade 1 may benefit from a less intensive follow-up, as they rarely progress to high grade cervical intraepithelial neoplasia grade 2 or 3 [10]. To date, few studies have examined the utility of p16INK4a immunohistochemical staining for the assessment of cervical biopsies in an African pathology setting [11,12]. We aimed to determine the inter-observer agreement for hematoxylin and eosin-stained uterine cervical biopsies and assess the utility of p16INK4a expression in the evaluation of these biopsies in Nairobi, Kenya.

## Methods

### Eligibility criteria

This study includes cervical biopsy cases reported as either negative for dysplasia, cervical intraepithelial neoplasia grade 1, 2, or 3 (including carcinoma in situ) at the Kenyatta National Hospital histology laboratory, Nairobi, Kenya, from June 2011 to June 2013. Patients with invasive cervical cancer were not included. We reviewed the original pathology slide, if available, or prepared new slides from the corresponding paraffin embedded block. Paraffin embedded blocks were retrieved from the histology archives at the study site using the laboratory numbers on the surgical pathology reports. Only well-preserved blocks containing a mucosal layer were included in the study. A total of 91 cases met the inclusion criteria and were transported to the University of Nairobi histology laboratory where hematoxylin and eosin and immunohistochemical staining was performed. Ethical approval for this study has been obtained from the Kenyatta National Hospital/University of Nairobi-Ethics and Research Committee (P15/01/2013).

### Specimen processing and interpretation

All samples were stained with hematoxylin and eosin using the standard staining procedure [13] and for p16INK4a expression using the manual immunohistochemical staining procedure as described by the manufacturer (Ventana Medical System Inc., Tucson, Arizona, United States). Two positive and two negative controls for p16INK4a were included in each testing batch and were subjected to the same test conditions as the study cases. The p16INK4a-stained slides were classified into three different patterns: negative, focal, and diffuse staining. Negative staining was defined as non-immuno-reactive, focal staining was defined as non-continuous staining of isolated cells or small cell clusters, and diffuse staining was defined as a continuous staining of cells with p16INK4a expression being nuclear as well as cytoplasmic. Only cases with diffuse staining were considered as positive for p16INK4a in subsequent analyses. All hematoxylin and eosin and p16INK4a-stained slides were independently reviewed by two separate, expert pathologists. Cases with discordant results were reviewed by a third pathologist who served as a tiebreaker. For study comparisons, the original diagnosis provided by the sign-out pathologist between June 2011 and June 2013 is referred to as the “primary” diagnosis, and the consensus histologic diagnosis obtained by the pathology study team is referred to as the “consensus review” diagnosis.

### Statistical analysis

We used 4x4 tables based on the cervical intraepithelial neoplasia system to compare the histologic diagnoses of the primary and the consensus review results. We then produced 2x2 tables based on the Bethesda system (low-grade squamous intraepithelial lesion versus high-grade squamous intraepithelial lesion) and calculated the unweighted kappa value to assess the degree of agreement between primary and consensus review results taking into account agreement by chance. Kappa values can range from -1 to +1 and the following interpretation is commonly used: ≤0, no agreement; 0.01 to 0.20, slight agreement; 0.21 to 0.40, fair agreement; 0.41 to 0.60, moderate agreement; 0.61 to 0.80, substantial agreement; and 0.81 to 1.00 as almost perfect agreement [14]. We used the Chi-squared test for trend to assess whether p16INK4a positivity increased with increasing severity of cervical lesion grade.

### Funding

This study was supported by an administrative supplement to P30 Cancer Centers Support Grants to Stimulate Collaborative Human Papillomavirus Malignancy Research in Low to Middle-Income Countries (CA016086). Dr. Zawadi Muvunyi was supported by a D43 International Research Training Grant (1D43CA153793, Principal investigator: Anastos) from the National Institutes of Health. Dr. Rohner was supported by a grant from the Swiss Cancer Research foundation (BIL KFS-4423-02-2018). The content of this publication is solely the responsibility of the authors and does not necessarily represent the official views of the funders.

## Results

Patients´ age ranged from 21 to 65 years (mean ± standard deviation: 40.2 ± 10.2 years). Information on histologic diagnoses and immunohistochemical staining patterns of cervical specimens was available for all 91 patients. There was substantial variability between primary and consensus review histologic diagnoses (Table 1). The most common primary diagnosis based on the hematoxylin and eosin-stained sections were cervical intraepithelial neoplasia grade 2 (n = 37; 40.7%) and grade 1 (n = 23; 25.3%). On consensus review, most cases were either negative for dysplasia (n = 32; 35.2%) or cervical intraepithelial neoplasia grade 3 (n = 24; 26.4%). With dichotomous categorization of cases into those with low-grade and high-grade squamous intraepithelial lesions, there was only moderate agreement between the primary and review histology results (kappa: 0.568, Table 2). Consensus review results confirmed most primary results that were negative for dysplasia (15/16, 94%) and cervical intraepithelial neoplasia grade 3 (12/15, 80%). However, there was substantially more disagreement for cervical intraepithelial neoplasia grade 1 cases with only 5/23 (22%) primary results confirmed upon consensus diagnosis, and for cervical intraepithelial neoplasia grade 2 with only 11/37 (30%) primary results confirmed. Of 39 primary results classified as low-grade squamous intraepithelial lesion, 90% (35/39) were confirmed by the consensus review. In contrast, 36 out of 52 (70%) primary high-grade squamous intraepithelial lesions were confirmed by consensus review.

**Table 1:** comparison of primary and consensus review histologic diagnoses among 91 histology readings in Nairobi, Kenya

		Consensus review pathology results				
		No dysplasia	CIN 1	CIN 2	CIN 3	Total
**Primary pathology results**	No dysplasia	15 (16%)	1 (1%)	0 (0%)	0 (0%)	16 (18%)
	CIN 1	14 (15%)	5 (5%)	4 (4%)	0 (0%)	23 (25%)
	CIN 2	1 (1%)	13 (14%)	11 (12%)	12 (13%)	37 (41%)
	CIN 3	2 (2%)	0 (0%)	1 (1%)	12 (13%)	15 (16%)
	Total	32 (35%)	19 (21%)	16 (18%)	24 (26%)	91 (100%)

Data are n (%); cell percentages shown. CIN, cervical intraepithelial neoplasia

**Table 2:** agreement between dichotomized primary and consensus review histologic diagnoses

		Consensus review pathology results		Cohen´s Kappa
		LSIL	HSIL	
**Primary Pathology Results**	LSIL	35 (38%)	4 (4%)	0.568
	HSIL	16 (18%)	36 (40%)	

Data are n (%). HSIL, high-grade intraepithelial lesion; LSIL, low-grade intraepithelial lesion

**Figure 1:**
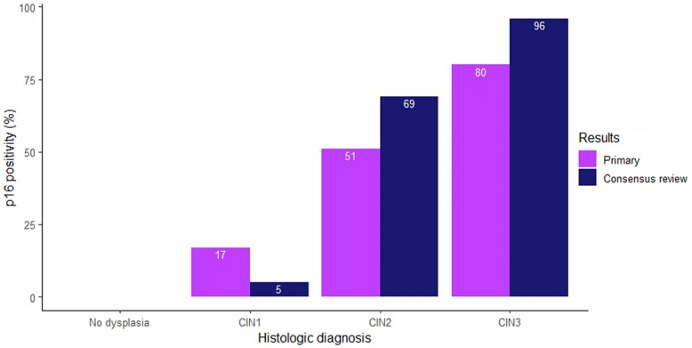
p16INK4a positivity by histologic diagnosis in primary and consensus review results

Overall, less than half of the cases demonstrated p16INK4a expression (n = 35; 38.5%). On primary diagnosis, p16INK4a expression increased with increasing severity of cervical lesion grade (p<0.001, Table 3). P16INK4a expression was observed in no cases negative for dysplasia (0%) and in 18% (4/23) of cervical intraepithelial neoplasia grade 1 cases. However, half (51%) of cervical intraepithelial neoplasia grade 2 cases and 80% (12/15) of grade 3 cases expressed p16INK4a (Figure 1). On consensus review diagnosis, p16INK4a positivity increased even more clearly with increasing severity of cervical lesion grade from 0% in cases negative for dysplasia, 5% (1/19) in cervical intraepithelial neoplasia grade 1 to 69% (11/16) in grade 2 and 96% (23/24) in grade 3 (p < 0.001, Table 3).

**Table 3:** p16INK4a expression stratified by histologic diagnosis among primary and consensus review pathology results

			p16INK4a		
		Frequency	Negative	Positive	p-value for trend
**Primary Pathology Results**	Negative	16	16 (100%)	0 (0%)	<0.001
	CIN 1	23	19 (83%)	4 (17%)	
	CIN 2	37	18 (49%)	19 (51%)	
	CIN 3	15	3 (20%)	12 (80%)	
**Consensus Review Pathology Results**	Negative	32	32 (57%)	0 (0%)	<0.001
	CIN 1	19	18 (95%)	1 (5%)	
	CIN 2	16	5 (31%)	11 (69%)	
	CIN 3	24	1 (4%)	23 (96%)	

Data are n (%); p-values from chi-squared test for trend. CIN, cervical intraepithelial neoplasia

## Discussion

We found significant variability in the histologic diagnoses of 91 cervical biopsies on hematoxylin and eosin with only moderate agreement (kappa: 0.568) between primary and final consensus review diagnoses. To improve the quality of pathology readings, we added p16INK4a immunohistochemical staining in a second step and found p16INK4a positivity to increase with the degree of cervical neoplasia in both primary and consensus review results. Our results showed a more notable increase in p16INK4a positivity in consensus review diagnoses, providing novel evidence of the utility of p16INK4a to improve cervical diagnosis in the context of an African pathology setting.

Our results are in line with previous studies showing only moderate inter-observer agreement for the interpretation of hematoxylin and eosin-stained cervical biopsies with kappa values ranging from 0.44 to 0.57 [15-17]. Some previous studies found even poorer inter-observer agreement for hematoxylin and eosin-stained biopsies [18,19]. The reproducibility of histologic diagnoses seems to be especially challenging for low grade cervical intraepithelial neoplasia grade 1 lesions [5]. In our study, more than half of the primary cervical intraepithelial neoplasia grade 1 diagnoses (14/23, 61%) were downgraded to “no dysplasia” upon the consensus review. This suggests that the pathologists who initially reviewed the cases had considerable difficulty in distinguishing cervical intraepithelial neoplasia grade 1 from reactive proliferations of the cervical squamous epithelium, including cervicitis, basal cell hyperplasia, and viral changes. This may also be attributed to the nuclear enlargement seen in inflammation or the immature appearance of squamous metaplasia being confused with dysplastic changes, such as increased nuclear to cytoplasmic ratio.

On consensus review, we downgraded 14 of 37 primary cervical intraepithelial neoplasia grade 2 cases (38%) to grade 1 or “no dysplasia”, and 2 cases initially interpreted as cervical intraepithelial neoplasia grade 3 were downgraded to “no dysplasia”. The overcalled diagnoses may be attributed to tangential orientation, squamous metaplasia, mucosal atrophy, and/or atypical reactive changes. However, it is also possible that the original lesion was cut through and no longer present on the slide which underwent review, as it has been shown that the diagnoses on cervical biopsies can vary significantly among histologic levels [20]. One could argue that the misinterpretations may simply reflect deficiencies in the diagnostic ability or training of the review pathologists; however, all of these pathologists were active in routine diagnostic histopathology and had considerable experience. An alternative and more likely explanation may be the lack of reproducibility of the morphologic criteria used to diagnose cervical intraepithelial neoplasia in general.

The histopathological interpretation of cervical biopsy specimens guides the subsequent management of women who have been screened for cytological abnormalities or high-risk human papillomavirus infections and have been referred for colposcopy for further diagnostic evaluation according to national screening guidelines. Diagnoses of high-grade squamous intraepithelial lesion (cervical intraepithelial neoplasia grade 2 or 3) lead to excisional or ablative therapeutic interventions to remove the abnormal tissue and prevent potential progression to invasive cancerous growth. Therefore, accuracy in distinguishing low-grade squamous intraepithelial lesions from high-grade squamous intraepithelial lesions is essential for avoiding overtreatment of false-positive cases and the undertreatment of false-negative cases. The distinction is also clinically relevant because many low-grade lesions will spontaneously regress [21], and excisional procedures potentially have a negative impact on reproductive outcomes [22].

While p16INK4a is not expressed in the normal epithelium, it is overexpressed in almost all cases of epithelial neoplasia of the uterine cervix because E7 protein of high-risk human papillomavirus inactivates retinoblastoma protein which normally inhibits the transcription of p16INK4a [23]. In our study, the degree of p16INK4a expression correlated well with the degree of cervical neoplasia, consistent with similar observations made in previous studies [11,24]. For example, a study from Tunisia, Africa, examined 87 cervical specimens for p16INK4a expression and found that none of the normal tissue and benign lesion samples were p16INK4a positive, but 50% of cervical intraepithelial neoplasia grade 1 and all cervical intraepithelial neoplasia grade 2+ cases expressed p16INK4a [11]. Research from Sudan, Africa, found p16INK4a positivity in 71 of 77 (92%) squamous cell carcinoma samples [12].

In our study, there was also no p16INK4a expression in lesions negative for dysplasia, based on either primary or consensus review results. For cervical intraepithelial neoplasia grade 1, approximately 17% of primary and 5% of consensus review slides showed diffuse p16INK4a expression. 75% of cervical intraepithelial neoplasia grade 1 cases which were upgraded to grade 2 during consensus review expressed p16INK4a. These findings demonstrate the role of p16INK4a in increasing diagnostic accuracy and as a marker of high-grade squamous intraepithelial lesion. Positivity for p16INK4a in consensus review cervical intraepithelial neoplasia grade 1 lesions was low (5%) as compared to other studies which reported p16INK4a positivity of 50% to 60% in grade 1 cases [11,25,26]. It is possible that the lower expression in our study was due to poor preservation of our blocks or to the relatively small number of cervical intraepithelial neoplasia grade 1 cases. The generally relatively high human immunodeficiency virus prevalence in our patient population might also play a role, as a lower percentage of p16INK4a positive cells in the cervices from high human immunodeficiency virus positive patients has been reported [27].

## Conclusion

We found substantial variability in interpretation of cervical biopsies based on hematoxylin and eosin staining alone. However, additional p16INK4a immunostaining appeared to notably improve diagnostic accuracy and reproducibility of cervical biopsy interpretations, as p16INK4a positivity was positively associated with the increasing severity of cervical lesion grade.

### What is known about this topic

- The burden of cervical cancer is high in Kenya;
- Diagnostic interpretation of hematoxylin and eosin-stained cervical tissue is subject to substantial discordance among pathologists;
- Immunohistochemical staining for p16INK4a can improve the inter-observer reproducibility of histologic diagnoses of cervical intraepithelial neoplasia.

### What this study adds

- We found large variability in histologic diagnoses of hematoxylin and eosin-stained cervical biopsies;
- p16INK4a positivity increased with the degree of cervical neoplasia;
- p16INK4a staining is a viable option to improve cervical diagnoses in a Kenyan pathology setting.
